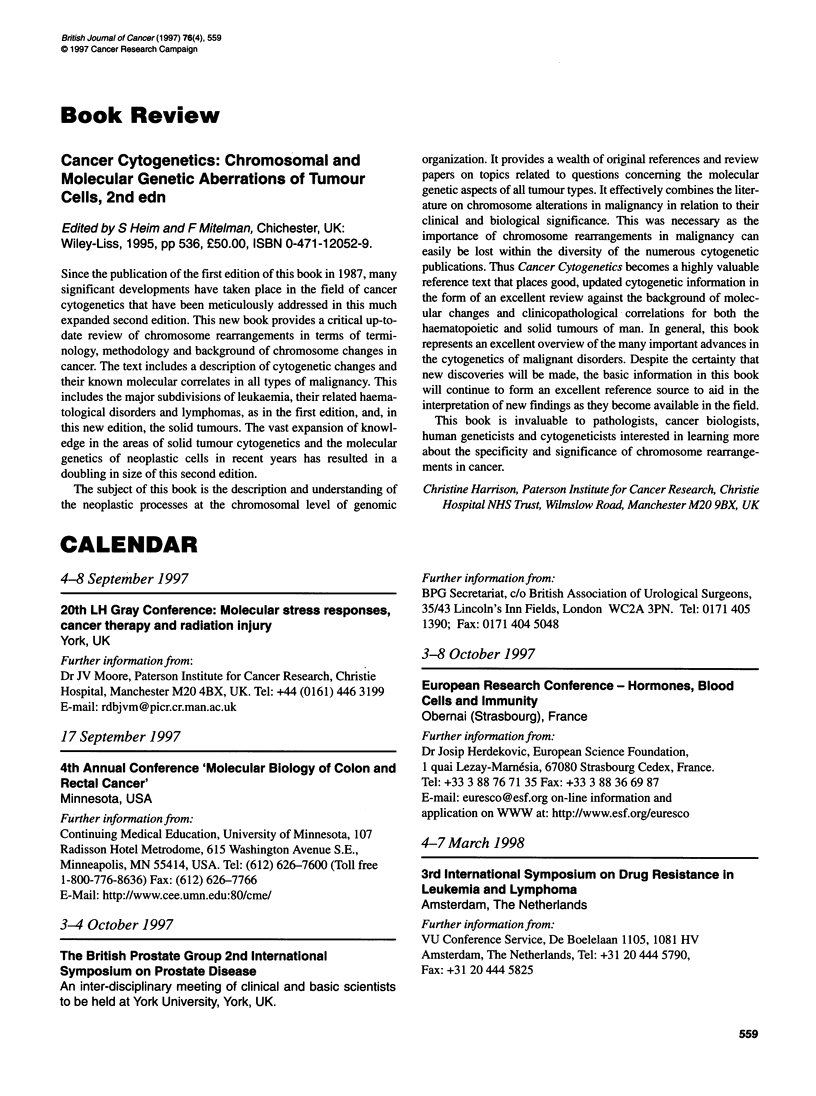# Cancer Cytogenetics: Chromosomal and Molecular Genetic Aberrations of Tumour Cells, 2nd edn

**Published:** 1997

**Authors:** Christine Harrison


British Journal of Cancer (1997) 76(4), 559
© 1997 Cancer Research Campaign

### Book Review

### Cancer Cytogenetics: Chromosomal and Molecular Genetic Aberrations of Tumour Cells, 2nd edn

*Edited by S Heim and F Mitelman,* Chichester, UK: Wiley-Liss, 1995, pp 536, £50.00, ISBN 0-471-12052-9.

Since the publication of the first edition of this book in 1987, many significant developments have taken place in the field of cancer cytogenetics that have been meticulously addressed in this much expanded second edition. This new book provides a critical up-to-date review of chromosome rearrangements in terms of terminology, methodology and background of chromosome changes in cancer. The text includes a description of cytogenetic changes and their known molecular correlates in all types of malignancy. This includes the major subdivisions of leukaemia, their related haematological disorders and lymphomas, as in the first edition, and, in this new edition, the solid tumours. The vast expansion of knowledge in the areas of solid tumour cytogenetics and the molecular genetics of neoplastic cells in recent years has resulted in a doubling in size of this second edition.

The subject of this book is the description and understanding of the neoplastic processes at the chromosomal level of genomic organization. It provides a wealth of original references and review papers on topics related to questions concerning the molecular genetic aspects of all tumour types. It effectively combines the literature on chromosome alterations in malignancy in relation to their clinical and biological significance. This was necessary as the importance of chromosome rearrangements in malignancy can easily be lost within the diversity of the numerous cytogenetic publications. Thus *Cancer Cytogenetics* becomes a highly valuable reference text that places good, updated cytogenetic information in the form of an excellent review against the background of molecular changes and clinicopathological correlations for both the haematopoietic and solid tumours of man. In general, this book represents an excellent overview of the many important advances in the cytogenetics of malignant disorders. Despite the certainty that new discoveries will be made, the basic information in this book will continue to form an excellent reference source to aid in the interpretation of new findings as they become available in the field.

This book is invaluable to pathologists, cancer biologists, human geneticists and cytogeneticists interested in learning more about the specificity and significance of chromosome rearrangements in cancer.

*Christine Harrison, Paterson Institute for Cancer Research, Christie Hospital NHS Trust, Wilmslow Road, Manchester M20 9BX, UK*